# Analysis of misdiagnosis of pancreatic acinar cell carcinoma with significantly elevated alpha-fetoprotein: a case report and literature review

**DOI:** 10.3389/fonc.2026.1795103

**Published:** 2026-07-16

**Authors:** Huihang Mei, Hao Li, Wenting Shang, Geng Yu, Mingjian Li, Yongbiao Ma

**Affiliations:** 1 School of Clinical Medicine, Shandong Second Medical University, Weifang, Shandong, China; 2 Weifang People’s Hospital, Department of Pancreatic surgery, Weifang, Shandong, China; 3 School of Medical Laboratory, Shandong Second Medical University, Weifang, Shandong, China

**Keywords:** acinar cell carcinoma, alpha-fetoprotein, multidisciplinary team, pancreaticoduodenectomy, solid pseudopapillary neoplasm

## Abstract

Acinar cell carcinoma (ACC) is a rare malignant exocrine tumor of the pancreas, often presenting with nonspecific symptoms that lead to diagnostic confusion with other pancreatic neoplasms. This article reports a case of a 72-year-old female ACC patient who was misdiagnosed as Solid pseudopapillary neoplasms (SPN) by an external hospital. Laboratory investigations upon admission revealed a markedly elevated serum alpha-fetoprotein (AFP) level of 2321.18 ng/mL, while contrast-enhanced CT of the upper abdomen demonstrated a mass in the pancreatic head, with the liver showing normal parenchyma and no focal masses or lesions. Following a multidisciplinary team (MDT) discussion, the patient underwent a radical Pancreaticoduodenectomy. The final histopathological diagnosis confirmed ACC. This case underscores that in patients with pancreatic masses exhibiting misleading imaging features suggestive of SPN, a significantly elevated serum AFP level should serve as a critical diagnostic clue prompting consideration of ACC. Integrating tumor marker analysis, imaging findings, and MDT evaluation is essential to minimize preoperative misdiagnosis of such rare entities.

## 1 Introduction

Acinar cell carcinoma (ACC) is a rare malignant exocrine tumor of the pancreas, primarily originating from pancreatic acinar cells and accounting for approximately 1% of all pancreatic tumors (1). This disease predominantly affects middle-aged and elderly males, with the pancreatic head being the most common site of origin. The male-to-female ratio is approximately 3.6:1 (2–4). Since Berner’s initial report in 1908, the pathogenesis of this condition remains incompletely understood. Current research suggests that genetic abnormalities and chromosomal instability may contribute to its development (5). The clinical manifestations of ACC lack specificity. In the early stages, it often presents with non-specific gastrointestinal symptoms such as abdominal pain and weight loss, which are easily overlooked or delayed. Consequently, approximately half of patients are already in the intermediate or advanced stages at the time of diagnosis (6, 7). It is worth noting that some patients may present with elevated serum alpha-fetoprotein (AFP) levels. Although uncommon, this can serve as one of the potential laboratory indicators suggesting ACC (5, 8, 9). Imaging evaluation is the primary step in differential diagnosis. While contrast-enhanced CT and MRI can clearly display tumor morphology, their findings often resemble those of pancreatic ductal adenocarcinoma (PDAC) and Solid pseudopapillary neoplasm (SPN), thereby increasing the risk of preoperative misdiagnosis. Currently, radical surgery remains the primary treatment modality, but postoperative adjuvant therapy still lacks standardized protocols based on high-level evidence (2). Due to the rarity of this disease, standardized diagnostic and treatment guidelines supported by large-scale, multicenter studies remain lacking. Therefore, systematic summarization and exchange of clinical experience are particularly crucial. This article reports a case of a female patient with ACC who presented with significantly elevated AFP levels, initially misdiagnosed preoperatively but confirmed pathologically postoperatively. Integrating the latest research advances, this report aims to enhance understanding of the diagnosis and management of this disease and provide reference for clinical practice.

## 2 Case report

A 72-year-old female patient visited a local hospital due to ‘upper abdominal pain for more than one month. An enhanced computed tomography (CT) scan of the upper abdomen showed a space-occupying lesion in the head of the pancreas, raising suspicion for a solid pseudopapillary neoplasm. For further diagnosis and definitive surgical treatment, she came to our hospital (Weifang People’s Hospital) for consultation. The patient had no previous medical history. Physical examination after admission revealed mild tenderness in the upper abdomen without rebound tenderness. No abdominal mass was palpable, and there was no obvious jaundice or significant enlargement of superficial lymph nodes. The results of admission laboratory tests were as follows:

• Complete blood count:White blood cells: 7.57×10^9^/L, Neutrophil percentage: 73.6%, Lymphocyte percentage: 18.4%, Red blood cells: 4.87×10^12^/L, Platelets: 290×10^9^/L, Procalcitonin:0.04µg/L
• Albumin transaminase 14 U/L, glutamic-oxaloacetic transaminase 18 U/L, Alkaline phosphatase 137 U/L, gamma-glutamyl transpeptidase 22 U/L, total bilirubin 17.7 µmol/L, direct bilirubin 5.9 µmol/L, indirect bilirubin 11.8 µmol/L, albumin 47.7g/L.
• Alpha-fetoprotein 2321.18 ng/mL, CA19-9 49.54 U/mL.
• Non-contrast and contrast-enhanced CT scans of the upper abdomen demonstrated mass in the pancreatic head. Multiple enlarged lymph nodes are noted in the abdominal cavity and retroperitoneum. The liver was normal in size and shape with smooth contours, and no abnormal parenchymal densities or enhancing lesions were observed. (**Figure 1**)

**Figure 1:**
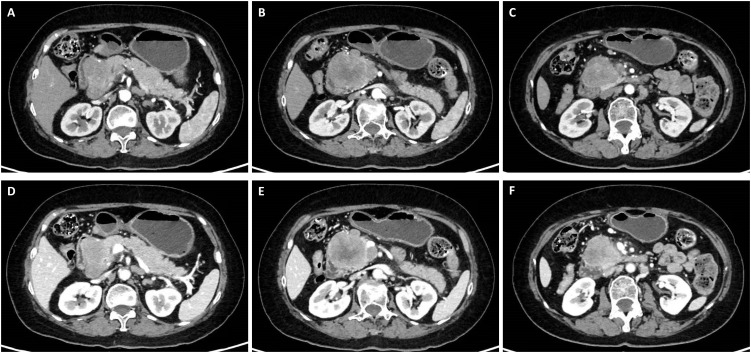
abdominal enhanced CT showed a mass in the head of the pancreas and multiple enlarged abdominal and retroperitoneal lymph nodes. The liver was normal in size and shape, with smooth margins, and no abnormal density or enhancement foci in the parenchyma **(A–C)** shows the arterial phase; **(D–F)** shows portal venous phase.

Based on the above examination results, a preliminary diagnosis of malignant pancreatic tumor was established. Following a multidisciplinary team (MDT) consultation at the hospital and in accordance with the “Practical Guidelines for the Diagnosis and Treatment of Pancreatic Cancer” issued by the Pancreatic Surgery Working Group of the Chinese Medical Association’s Surgery Branch, the patient was determined to have clear surgical indications. Preoperative assessment revealed no absolute contraindications for surgery. Consequently, we have decided to proceed with surgical treatment for the patient.

After obtaining informed consent from the patient and family members and signing the relevant documents, we performed a radical Pancreaticoduodenectomy. The surgery lasted approximately 300 minutes with blood loss of about 300 ml. During the procedure, the tumor was located in the pancreatic head, measuring approximately 5×5 cm. Enlarged lymph nodes were visible in the surrounding area, but no obvious metastatic lesions were found in the liver, omentum, or abdominal wall. The Postoperative pathological results indicated malignant pancreatic tumor involving the peripancreatic fat, the distal common bile duct, and the muscularis mucosae of the duodenum. A total of 16 lymph nodes were cleared, with tumor metastasis found in 6 of them. Immunohistochemical staining results: BCL10 (+), AFP (+), β- Catenin (membrane +), CgA (-), syn (8% positive cells), ckpan (+), ck8/18 (+), CK7 (+), CK19 (partial+), DPC4 (+), AAT (+), villin (+), Ki-67 proliferation index was about 40% (**Figure 2**). After consulting with the pathologists and considering the immunohistochemistry results, the final diagnosis was pancreatic acinar cell carcinoma.

**Figure 2:**
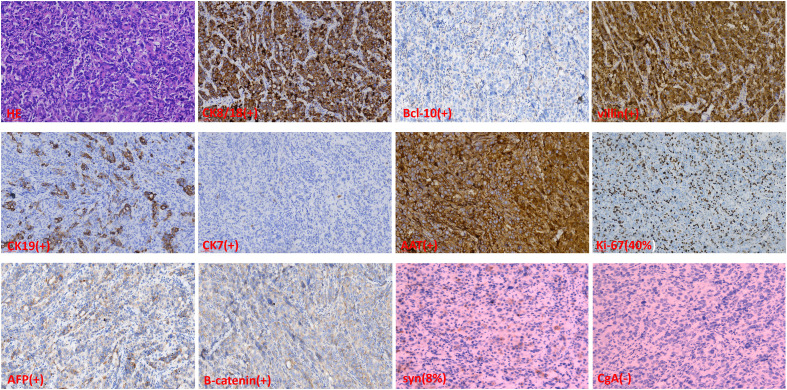
Morphologically, ACC has distinct acinar differentiation characteristics, typically showing acinar arrangement(HE × 200). Immunohistochemical staining showed that the tumor cells were positive for BCL10, AFP, β- Catenin (membranous), CKpan, CK-8/18, CK-7, CK-19, AAT and Villin, while CgA was negative. Syn was focally positive in 8% of tumor cells. The Ki-67 proliferation index was approximately 40% (immunohistochemistry × 200).

The patient had an uneventful postoperative course and was discharged on the 13th postoperative day. Following discharge, we initiated chemotherapy using the gemcitabine plus albumin-bound paclitaxel (AG) regimen. The first chemotherapy cycle commenced one month postoperatively, with follow-up examinations every three months. The patient has now completed four cycles of chemotherapy.

## 3 Discussion

This case involves a patient initially misdiagnosed at another hospital with a solid pseudopapillary neoplasm (SPN)of the pancreas, who was ultimately diagnosed with acinar cell carcinoma (ACC) and achieved favorable treatment outcomes. The differential diagnosis approach and individualized treatment experience reflected in the diagnostic and therapeutic process can provide a reference for the clinical management of rare ACC. The clinical manifestations of acinar cell carcinoma (ACC) of the pancreas lack specificity. Common symptoms include abdominal pain (approximately 60%), weight loss (approximately 45%), and vomiting (approximately 20%), while jaundice is relatively uncommon (approximately 12%) (10). This characteristic stands in sharp contrast to pancreatic ductal adenocarcinoma (PDAC), which often involves the common bile duct due to its invasive growth, resulting in a significantly higher incidence of jaundice (about 55%) (11, 12). In this case, the patient only presented with abdominal pain and did not develop jaundice, which aligns with the common clinical pattern of ACC. The lower incidence of jaundice in ACC is associated with its growth pattern, as the tumor typically exhibits “expansive” rather than “invasive” growth, often displacing rather than directly invading the common bile duct (13). Therefore, for pancreatic head lesions without overt jaundice, the significant difference in jaundice incidence between ACC and PDAC provides an important clue for their preliminary differentiation.

In addition to clinical manifestations, the patient’s significantly elevated preoperative alpha-fetoprotein (AFP) level (2321.18 ng/mL) provided a crucial diagnostic clue. After excluding more common causes such as liver cancer and germ cell tumors through imaging and intraoperative exploration, the cause of its elevation is clearly attributed to pancreatic origin. This result is completely opposite to the typical presentation of SPN. SPN predominantly affects young women, typically presents with negative serum AFP levels, and rarely involves lymph node metastasis (14). In contrast, this patient has both significant elevation of AFP and lymph node metastasis characteristics. This contradiction strongly suggests inaccurate initial diagnosis, prompting us to include rare tumors such as pancreatic acinar cell carcinoma (ACC) in the scope of differential diagnosis. To further characterize the occurrence of this distinct clinical phenotype, we conducted a literature search and summarized previously reported cases of ACC associated with elevated AFP from both domestic and international studies in recent years. The detailed case information is presented in **Table 1**. In terms of age distribution, the patients ranged from 10 to 79 years, with AFP levels varying from 71.5 ng/mL to 32,000 ng/mL, indicating significant clinical heterogeneity of this phenotype. There are significant differences in the background of onset, clinical symptoms, laboratory tests, etc. among the cases, but they are all accompanied by varying degrees of elevation in AFP.

**Table 1:** Summary of cases of pancreatic acinar cell carcinoma with elevated AFP.

Author	Year	Age	Sex	Primary diagnosis	Main symptoms	AFP(ng/mL)	Treatment methods	Clinical outcome
Eriguchi et al. (15)	2000	67	M	Rupture of HCC	Severe upper abdominal pain	125.5	Distal pancreatectomy combined with splenectomy	NA
Chen et al. (16)	2001	78	F	ACC	Upper abdominal pain, nausea	8164	Chemotherapy (Gemcitabine)	NA
Mori et al. (17)	2004	33	M	ACC	Upper abdomen, back pain.	12886	Chemotherapy (5-FU and CDDP)	NA
Mueller et al. (18)	2005	19	M	ACC	Stomach pain for 6 weeks	1219	Combined radiotherapy and 5-FU regimen	Died(18M)
Illyés et al. (19)	2007	10	M	ACC-associated Cushing’s syndrome	Abdominal pain, bone pain	7664	Chemotherapy (NA)	Died(24M)
Kolb-van Harten et al. (20)	2007	77	M	ACC	Increasing fatigue and impaired appetite	32000	Chemotherapy (Gemcitabine and mitomycin C)	Died(7M)
Lin et al. (21)	2007	47	M	ACC	No obvious symptoms	463.69	distal pancre atectomy and splenectomy	No recurrence observed
Fujii et al. (22)	2010	59	M	ACC	Anemia	8228	Pancreaticoduodenectomy	Died (36M)
Chung et al. (23)	2011	72	F	ACC with fatty change.	No obvious symptoms	1945	Pancreaticoduodenectomy	No recurrence observed
Ellerkamp et al. (24)	2012	79	M	ACC	Upper abdominal pain	7979	Pancreaticoduodenectomy	Died(2M)
Hiraoka et al. (25)	2012	25	F	ACC(Preoperative misdiagnosis of hepatocellular carcinoma)	Huge abdominal tumor	1222.4	Pancreaticoduodenectomy	NA
Kim et al. (26)	2014	72	F	ACC	No obvious symptoms	2254.1	Pancreaticoduodenectomy	Died(30M)
Morikawa et al. (27)	2016	12	F	ACC	Upper abdominal pain	173.1	Pancreaticoduodenectomy	No recurrence observed
Valente et al. (28)	2016	30	M	ACC(with liver metastasis and retroperitoneal metastasis)	Abdominal pain	17373	Chemotherapy (NA)	NA
Zhang et al. (1)	2018	69	M	ACC	Jaundice of the skin and sclera accompanied by upper abdominal pain	71.5	Pancreaticoduodenectomy(started gemcitabine chemotherapy regimen 1 month after surgery)	No recurrence observed
Busch et al. (29)	2020	27	M	ACC(with BRAFV600E-mutated)	Abdominal pain, weight loss	1358.9	FOLFIRINOX chemotherapy	Died(21M)
Qin et al. (30)	2021	48	F	Metastatic pancreatic acinar cell carcinoma	No obvious symptoms	1103.4	Lenvatinib combined with Sintilimab therapy	Alive(21M)
Zhao et al. (31)	2023	44	F	ACC (with distant metastasis)	Back pain	109.3	Multimodal treatment (chemotherapy, targeted therapy, immunotherapy)	Alive(60M)

M:male, F:female, NA:not available, ACC: pancreatic acinar cell carcinoma, AFP:alpha-fetoprotein, CDDP:cisplatin, 5-FU:5-fluorouracil, M:months.

Imaging examination is a key tool for preliminary assessment of pancreatic space occupying lesions. ACC usually presents as a solid or cystic mass with clear boundaries and large volume on enhanced CT or MRI, with common necrosis or cystic changes inside. After enhancement, it shows uneven enhancement, and the degree of enhancement is often lower than that of normal pancreatic parenchyma (32). However, this imaging feature is not unique to ACC, and PDAC often presents as an infiltrative mass with blurred boundaries and lack of blood supply, often accompanied by “double tube sign” and vascular invasion (33). SPN is characterized by a well-circumscribed, encapsulated mass. It often demonstrates mixed cystic and solid components, with possible calcification and hemorrhage. On contrast-enhanced imaging, the solid portions, capsule, and internal septa exhibit progressive enhancement (34). In addition, pancreatoblastoma (PB), although more common in children, can also occur in adult patients. It typically presents as an exophytic mass with well-defined borders, demonstrating progressive homogeneous enhancement on contrast-enhanced imaging, and may be accompanied by internal calcification and necrosis (35). Mixed acinar-neuroendocrine carcinoma (MANEC), due to its dual acinar and neuroendocrine components, exhibits more complex imaging features. Jakobsen et al. (36) reported a case of MANEC in the pancreatic tail, which exhibited imaging features of well-defined tumor margins, intact capsule, and hemorrhage visible in the cystic region. Due to the rarity of MANEC, no characteristic imaging features have been established, and it remains difficult to distinguish from ACC on imaging alone. The imaging findings in the present case illustrate this overlapping presentation and further highlight the limitations of relying solely on radiographic examination for diagnosis.

Pathological diagnosis is the gold standard for confirming ACC, with its core principle being the combined assessment of the tumor’s unique morphological features and immunohistochemical phenotype (37). At the morphological level, ACC exhibits distinct features of acinar differentiation, typically manifested as acinar like arrangement structure. Zymogen granules are visible within the cytoplasm of tumor cells, nuclei are distributed toward the basal pole, and a prominent single nucleolus is present (38). Immunohistochemical analysis provides further critical support for diagnosis, with ACC characterized by positive expression of markers associated with acinar differentiation. In recent years, BCL10 has been confirmed as a highly sensitive and specific marker of acinar differentiation, offering significant diagnostic value in ACC (38). The cytokeratin expression pattern of ACC also follows a typical pattern: CK8/18 is usually diffusely positive, whereas CK7 and CK19 are predominantly negative or show focal positivity (39). The pathological and immunohistochemical findings in this patient were consistent with the characteristic features of ACC. The tumor tissue exhibits coexistence of acinar differentiation areas and solid regions. Immunohistochemical detection revealed diffuse positivity for the specific acinar marker BCL10, while CK8/18, alpha-1-antitrypsin (AAT), Villin, and AFP were all positive. In contrast, CK7 was only focally positive and CK19 was partially positive. This forms a characteristic phenotypic pattern of “high expression of acinar markers and low expression of duct-related markers,” strongly supporting the diagnosis of ACC.

When establishing the diagnosis of ACC, it is essential to systematically exclude tumor types with overlapping histological features, especially mixed neuroendocrine non neuroendocrine tumors (MiNEN) (formerly known as MANEC) and pancreatoblastoma (PB). According to the WHO Classification of Tumors of the Digestive System (5th edition), the diagnosis of MiNEN requires that each component, both the neuroendocrine and the non-neuroendocrine component, account for at least 30% of the total tumor volume (40). In the present case, the highly specific neuroendocrine marker chromogranin A (CgA) was completely negative, while synaptophysin (Syn) only showed scattered weak positivity, with positive cells accounting for 8% of tumor cells, far from meeting the diagnostic criteria of MiNEN. Combined with the absence of characteristic organ like structures of neuroendocrine tumors in morphology, the diagnosis of MiNEN can be definitively excluded. In addition, the serum AFP of the patient in this case was significantly elevated. To further clarify its source, we performed immunohistochemical staining for AFP on the tumor tissue, which revealed diffuse cytoplasmic positivity in the tumor cells, confirming that the elevated serum AFP was indeed produced by tumor cells themselves. Although PB can also show elevated AFP, the key basis for its diagnosis lies in the characteristic squamous nest structure and β- catenin nuclear/plasma positive expression caused by Wnt pathway activation (32). In this case, a total of 10 tissue blocks were sampled, comprehensively covering the lesion. Pathological review by two independent pathologists revealed no squamous nest structures, and β-catenin demonstrated complete membranous positivity without nuclear or cytoplasmic accumulation, effectively excluding the diagnosis of PB. Through this systematic pathological evaluation, the case was ultimately diagnosed as ACC.

For the treatment of ACC, radical surgical resection remains the cornerstone for achieving potential cure and long-term survival in patients with localized or locally advanced ACC (2). The preoperative evaluation of this patient showed that the pancreatic head mass with lymph node enlargement, without distant metastasis, was still resettable. After the comprehensive evaluation of MDT, radical Pancreaticoduodenectomy was performed. Wisnoski et al (41)analyzed 672 patients with ACC and showed that the 5-year overall survival rate of patients who underwent surgical resection was significantly higher than that of patients who did not undergo surgery (71.6% vs 22%), with a median overall survival of 123 months. A Korean multicenter study also confirmed that the 5-year overall survival rate of patients after radical resection was 57.4%, and the median survival was 78.8 months (42). Given the high-risk pathological features in this patient, postoperative adjuvant therapy is necessary. Although no universally standardized adjuvant chemotherapy regimen exists for ACC, accumulating evidence suggests a survival benefit. Zhou et al. (8)demonstrated that postoperative adjuvant chemotherapy significantly prolonged median overall survival in ACC patients (37.7 months vs. 18.9 months, P = 0.01) and served as an independent prognostic protective factor. In the selection of chemotherapy regimens, the patient’s physical status is often considered. A systematic review by Glazer et al. (7) indicates that patients with good physical status are typically recommended to receive the FOLFOX or FOLFIRI regimens, whereas those with poorer physical status are more suitable for the gemcitabine plus albumin-bound paclitaxel (AG) regimen. In this case, the patient’s postoperative physical status was assessed as ECOG 2, and pathology revealed high-risk features including a Ki-67 proliferation index as high as 40%. After MDT discussion and thorough communication with the patient and family, it was ultimately decided to use the AG (gemcitabine + albumin paclitaxel) regimen for adjuvant chemotherapy.

On this basis, we developed a systematic postoperative follow-up and management plan for this patient. Due to the extreme rarity of ACC, no specific follow-up guidelines are currently available, and clinical practice generally follows the surveillance strategies established for PDAC. According to the consensus of the Pancreatic Disease Committee of the Society of Abdominal Radiology, we adopted the following follow-up schedule: every 3 months during the first postoperative year, every 3–6 months during the second and third years, and every 6–12 months thereafter (43). Each follow-up visit included a physical examination, routine blood tests, liver and renal function assessments, tumor marker measurements (CA19-9 and AFP), and contrast-enhanced CT or MRI of the chest and abdomen. In this case, the patient had significantly elevated preoperative AFP levels (2321.18 ng/mL). Immunohistochemical analysis confirmed diffuse AFP expression in tumor cells, indicating that AFP can serve as a specific marker for monitoring recurrence. Persistent elevation of AFP levels during follow-up, even in the absence of imaging findings, warrants a high suspicion of recurrence. In terms of long-term patient management, after the patient received AG regimen chemotherapy, a temporary decrease in white blood cell count occurred. Granulocyte colony-stimulating factor (G-CSF) was promptly administered, and the white blood cell count returned to the normal range, with no subsequent severe myelosuppression. Regarding nutritional support, the patient’s weight and nutritional status were regularly monitored, and his physical condition remained stable.

With the advancement of precision medicine, the molecular characteristics of ACC offer new perspectives for treatment. In contrast to PDAC, ACC rarely harbors mutations in KRAS or TP53. Instead, its characteristic alterations involve the Wnt/β-catenin pathway, BRAF/RAF1 rearrangements, and defects in DNA damage repair pathways (44). Emerging evidence indicates that ACC patients with homologous recombination defects may benefit from PARP inhibitor therapy, while specific gene rearrangements provide potential targets for targeted interventions in ACC (45, 46). This highlights the critical importance of molecular testing in developing personalized treatment strategies for ACC. During the diagnosis and treatment of this case, although we recommended the patient to complete molecular testing to identify potential opportunities for targeted therapy, the patient explicitly refused. This situation highlights the practical challenges still encountered in promoting ACC molecular testing in clinical practice. In the future, it is essential to strengthen patient communication and health education to improve their understanding of the clinical value of molecular testing. This will be crucial for translating the promise of precision medicine into tangible benefits in the management of ACC.

In summary, this case illustrates the complete diagnostic journey of an ACC with markedly elevated AFP, from initial misdiagnosis to final confirmation. It underscores the critical importance of vigilance toward atypical laboratory findings, such as a significantly elevated AFP. The discordance between the elevated AFP and the typical imaging features of a solid pseudopapillary neoplasm (SPN) was the key clue that corrected the misdiagnosis, while the characteristic immunohistochemical profile provided the definitive evidence for ACC. Treatment decisions should integrate pathological risk stratification, patient performance status, and evidence-based data. The patient’s favorable short-term outcome following comprehensive therapy highlights the value of an active, systematic treatment approach. Future efforts should focus on accumulating more clinical cases, conducting multi-omics research, and implementing molecularly stratified clinical trials to advance the precision development of the ACC diagnosis and treatment system.

## Data Availability

The original contributions presented in the study are included in the article/Supplementary Material. Further inquiries can be directed to the corresponding author.
